# Review on the Occurrence of Mycotoxigenic Fungi in Dried Fruits and the Role of Stored-Product Insects

**DOI:** 10.3390/toxins17070313

**Published:** 2025-06-20

**Authors:** Dimitrios-Evangelos Miliordos, Georgia V. Baliota, Christos G. Athanassiou, Pantelis I. Natskoulis

**Affiliations:** 1 Institute of Technology of Agricultural Products, Hellenic Agricultural Organization (ELGO)—“DIMITRA”, Sofokli Venizelou 1, 14123 Likovrisi, Greece; dim.miliordos@gmail.com; 2 Laboratory of Entomology and Agricultural Zoology, Department of Agriculture, Crop Production and Rural Environment, University of Thessaly, Phytokou Str., 38446 Volos, Greece; mpaliota@agr.uth.gr (G.V.B.); athanassiou@uth.gr (C.G.A.)

**Keywords:** fungi, mould, mycotoxins, pests, dried fruits, food safety

## Abstract

Dried fruits, which are widely produced in different parts of the world, and, especially in the Mediterranean basin, are broadly known for their durability and their nutritional value. This is primarily due to their ability to be stored for long periods of time and their concentrated nutrient content. However, these fruits can be at risk of contamination by specific stored-product insects and various toxigenic fungal species at different stages of their production process, including cultivation, harvesting, processing, drying, and storage. As a result, the dried fruits that are consumed may contain mycotoxins, which pose a potential risk for human health. The risk is significant in both industrialized and developing nations, as climate change and inadequate sanitation practices contribute to the proliferation of mycotoxins in these commodities. It is worth noting that there are several factors that contribute to the production of mycotoxins, such as the type of fruit, geographical location, climatic conditions, harvest treatments, and storage management practices, with specialized insects, known as “stored-product insects”, playing a crucial role in this latter stage. Therefore, it is critically important to gain a comprehensive understanding of the interaction among insects, fungi, and mycotoxins to effectively mitigate this problem. In this review, the primary objective is to bridge the knowledge gap by consolidating data from various regions to gain a global perspective on this topic.

## 1. Introduction

Fruits are a perishable type of food with a rather short life to reach the consumer’s table, with the potential to undergo dehydration and thus inhibit the microbial and enzymatic activity that leads to their spoilage. The process of dehydration can be carried out by either natural means, such as traditional sun drying, or through modern processes like the use of specialized dryers or dehydrators, wherein the water content is reduced to a level that ensures the desired outcome. They hold considerable significance for the economy as they engage perishable raw materials, thereby diminishing the expenses associated with packaging, handling, storage, and transportation. The methodologies employed in the processing and utilization of dried fruits present avenues for secure, prolonged storage, thereby enhancing the market valuation of these commodities. Indeed, the contemporary preservation techniques employed prolong their shelf life, facilitating their availability and distribution throughout the year in regions where they are not cultivated [1], without any substantial degradation of their nutritional value, which further contributes to their economic relevance. However, the drying process significantly affects the retention of nutrients in fruits, particularly sensitive compounds like vitamin C. While some macronutrients may remain stable, the degradation of soluble vitamins during drying is notable. Research indicates that various drying methods, including air, oven, and vacuum drying, lead to substantial losses of vitamin C and other bioactive compounds. Vitamin C is highly sensitive to heat and oxygen, resulting in significant losses during drying. For instance, oven drying has been shown to reduce vitamin C levels considerably compared to vacuum drying, which preserves more of this nutrient [2]. Different drying techniques yield varying retention rates of nutrients. Vacuum drying maintains higher concentrations of vitamin C and phenolic compounds compared to traditional methods [2,3]. Conversely, while drying can lead to nutrient losses, it also concentrates certain compounds, potentially enhancing the overall nutritional profile of dried fruits. This duality highlights the importance of optimizing drying methods to balance nutrient retention and product quality.

Moreover, they present a longer shelf life, allowing for increased availability to consumers [4]. Additionally, dried fruits can be used in the development of novel value-added products, such as instant soups, baking, dairy, and confectionery, which can contribute to the economy of the country [5]. They have been used since ancient times and have various health benefits, like reducing blood pressure and blood fat, resisting oxidation, enhancing immunity, and improving cardiovascular functions [6]. Moreover, dried fruits can contain protective compounds that are beneficial for human health [7].

They have significant nutritional importance due to their high content of minerals, proteins, and antioxidants, making them highly beneficial for human health [8]; at the same time, they are a good source of dietary fibre and provide a concentrated form of essential nutrients found in fresh fruits. Dried fruits such as dates, raisins, prunes, and Goji berries, have been found to be rich in protein, fat, dietary fibre, and minerals such as calcium, magnesium, and iron [9], being a convenient and healthy alternative to modern snacks of reduced nutritional value, providing a link between recommended fruit intake and actual consumption [8,10,11].

The contamination of mycotoxins is a notable source of decrease in food production, posing a potential danger to the health and safety of consumers. Due to their inherent structural durability and imperviousness to high temperatures, mycotoxins present a formidable challenge in terms of decontamination and detoxification during the process of food manufacturing, ultimately leading to their prolonged presence in various food items [12,13]. On the other hand, climate change is considered to further contribute to the presence and contamination of food by mycotoxins in Europe. The European Food Safety Authority (EFSA) predicts mostly harmful regional effects on mycotoxin occurrence [14]. Nevertheless, accurately forecasting the influence of climate change on the synthesis of mycotoxins is difficult due to the intricate interaction of multiple variables [15].

Mycotoxins are diverse substances produced by filamentous fungi. They have unique physicochemical properties due to their structural variation. Numerous types of mycotoxins are known, but only handfuls are of concern due to their considerable toxic effects, including carcinogenic, teratogenic, nephrotoxic, and hepatotoxic effects. Fungi of the genera *Aspergillus* and *Penicillium* can be found in various fruits, which are more vulnerable to fungal infections when ripen, due to pH increase, softened skin layers, increased carbohydrates, and weakened defence barriers [16]. Dried fruits, including grapes and figs, can also be infested. For this reason, the regulation of some mycotoxins in dried fruits is mandated by law. For instance, the EU has implemented strict rules to restrict the levels of ochratoxin A (OTA) and aflatoxin B1 (AFB1) in dried fruits [17,18,19,20]. Indicatively, the permitted maximum level (ML) for OTA in dried vine fruit is 10 µg kg^−1^, while that for AFB1 in dried fruits for human consumption or food ingredients is 2 µg kg^−1^ and that for total aflatoxins (AFB1 + AFB2 + AFG1 + AFG2) is 4 µg kg^−1^ [17,18]. A more recent EU regulation was established, setting the level of aflatoxins (AFs) in dried figs at 10 µg kg^−1^ [18]. In this context, it has been found that stored-product insects play a crucial role in mycotoxin contamination of different durable commodities at their post-harvest stages [21,22,23] and thus, control of these species is one of the cornerstones in the effort to mitigate this problem.

The aim of this review is to assess the aforementioned concerns related to the preservation and safety of dried fruits, taking into account their essential importance as food. The review encompasses the analysis of pest infestation, the biosynthesis of mycotoxins, and relative toxigenic fungi ecophysiology, taking into consideration the climate change effect, whilst presenting recent legislation and future perspectives. The ultimate objective of this review is to add knowledge and enhance understanding of stored-product insects’ interaction with mycotoxins and relative producing fungi, and thus promote food safety, quality control measures, and the development of novel dried fruit products.

## 2. Insects of Dried Fruits

Several arthropods can become serious post-harvest pests in dried fruits [24]. The potential origin of these infestations could be attributed to individuals of many insect orders, particularly Coleoptera, Diptera, Hymenoptera, and Lepidoptera. However, a vast majority of cases involving post-harvest contamination of dried fruits can be attributed to stored-product insects and mites that include more than 2000 species, mostly grouped into four taxa: Coleoptera, Lepidoptera, Psocoptera, and Acari [24,25,26,27]. In contrast to pests found in agricultural fields, these species have developed adaptations that enable them to thrive and reproduce in over 1.100 agricultural commodities [24]. Thus, wherever such products are produced, their major pests are the same species (Table 1) that have been distributed by the international trade of goods [25,28]. Understanding the nature of the species that cause damages to dried fruits is essential for developing effective control measures and to ensure future global food security [29].

The development rate, lifespan, and egg production vary among species and can be associated with the food availability and the thermal conditions within a given facility [24]. These, in general, are among the most important factors that determine annual generations and the overall population growth. The infestation pattern of stored dried fruits by such insects, as well as the identification of the infestation, is influenced to some extent by their behaviour. This includes their mating and aggregation patterns, dispersal tendencies, and preferences for laying eggs [30]. Nevertheless, the primary cause of damage to the product stems from the larval stages of beetles and moths [24]. It is important to note, however, that adult beetles also contribute to the degradation of the product by their active feeding behaviour, thereby having a substantial influence in this respect. Conversely, the larvae of moths produce a thin webbing which, in instances of severe infestation, appears as a delicate white membrane enveloping the entirety of the product. This phenomenon leads to costly rejections and market loss at the wholesale level as a result of quality concerns or compliance with phytosanitary regulations [31]. Most stored-product beetles possess the ability to fly and can be easily increase the infestation in wide areas, in a very short period of time [24,32]. Moreover, individuals of all taxa can easily infiltrate various goods either through airborne or ground-based means, enter storage facilities, or be transferred with dried fruits. Their small body size along with their cryptic behaviour enables them to find refuges in many sites within the storage facility and the machinery, while they are capable of entering even the best-designed and tightest packages of products, avoiding partially or completely treatments with residual insecticides, aerosols, or fumigants [24,30,33]. Nevertheless, most of the species that are major pests of dried fruits, including moth larvae and adult beetles, cannot penetrate undamaged kernels or packaging, making them secondary pests [34]. Generally, they benefit from finding and exploiting small packaging entry sites established by other insects (primary pests) to get into and infest goods [34,35].

Among the most common pest beetles of fruits are the sap beetles of the Nitidulidae family. The sap beetles can be found in the field but also during storage, are mostly saprophagous and mycetophagous, and are attracted to ripening and decaying fruits, but also by other fermented plant tissues such as mouldy grains and hay, bark of recently dead trees, and others [36,37,38]. Nonetheless, only a few of these beetles are regarded as economically important pests of stored products worldwide, affecting stored grain, dried fruits, oilseeds, cacao, and numerous other commodities [24]. Indicatively, the dried fruit beetle, *Carpophilus hemipterus* (L.), the Australian sap beetle, *Carpophilus davidsoni* Dobson, the confused sap beetle, *Carpophilus mutilatus* Erichson, and the pineapple beetle, *Carpophilus humeralis* (F.), pose a threat in the food industry when they develop in stored dried fruits [39,40]. These species can cause rapid and substantial mass loss by chewing the product. Moreover, they can serve as vectors for pathogens, carrying several bacteria and yeast cells, resulting in a significant decline in the quality of the stored product [41,42]. Although data regarding the occurrence and abundance of these species [43], the timing of biological events [40,44], and the organisms serving as hosts [39] can be found in the literature, the majority of these investigations are focused on adult populations, leaving a lack of data regarding the biological characteristics of immature life stages of *Carpophilus* spp. The study conducted by James and Vogele [45] offers a thorough examination of the egg-to-adult development and survivorship of three economically significant Carpophilus species, in relation to temperature. However, additional research is necessary to investigate the influence of all developmental stages of these species when infesting dried fruits and other stored products under various storage conditions.

The saw-toothed grain beetle, *Oryzaephilus surinamensis* (L.), and the merchant grain beetle, *Oryzaephilus mercator* (Fauvel) (Coleoptera: Silvanidae), along with the rust-red flour beetle, *Tribolium castaneum* (Herbst) (Coleoptera: Tenebrionidae), are also noteworthy secondary stored-product pests that have the potential to infest dried fruits [30,46]. These species have a worldwide presence and hold a serious pest status due to their wide-ranging host preferences, rendering them economically consequential within many stages of processed commodities distribution and storage systems [24]. *Oryzaephilus surinamensis* exhibits a preference for food items with elevated carbohydrate content and thus is frequently encountered in stored dried fruits, while *O. mercator* tends to be predominantly present on products that possess a greater oil content, hence making it more likely to be a concern on tree nuts and peanuts [47,48]. Their presence in dried fruits does not lead to significant weight loss. However, it does contribute to a substantial contamination of the goods due to the accumulation of frass (insect excrement) and body parts. On the other hand, *T. castaneum* has one of the highest population growth rates of all stored-product beetles due to its reproductive rate and long reproductive life [49]. Both adults and larvae feed on dried fruits. When infestation levels reach a critical level, they produce excessive moisture which promotes the proliferation of fungi, bacteria, and mites. Additionally, these insects release benzoquinones, a carcinogenic fluid with an unpleasant odour that renders the product unsuitable for consumption [31,50].

Several pyralid moth species have been identified as economically important secondary pests for dried fruits. These include the Indian meal moth, *Plodia interpunctella* (Hübner), the almond moth, *Cadra cautella* (Walker), the tobacco moth, *Ephestia elutella* (Hübner), the raisin moth, *Cadra figulilella* (Gregson), and the navel orange worm, *Amyelois transitella* (Walker) (Lepidoptera: Pyralidae). *Plodia interpunctella*, known as the most prevalent and extensively dispersed moth species in stored products, damages a wide range of goods [35]. According to Perez-Mendoza and Aguilera-Peña [51], this pest has the ability to infest several types of preserved grains and pulses, dried fruits and nuts, dried vegetables, and processed goods. The life cycle of *C. cautella* has similarities to that of *P. interpunctella*, yet with a preference for areas characterized by higher temperatures and increased humidity levels [52,53]. On the other hand, *E. elutella* is adapted to cooler climates and has a comparatively slower development rate under warmer settings when compared to *P. interpunctella* and *C. cautella* and hence, *E. elutella* is not commonly encountered in tropical regions [54]. *Cadra figulilella*, predominantly distributed in Mediterranean regions, as well as comparable climatic conditions in the Americas and Australia, is a pest found in agricultural fields, known for its tendency to target fruit that is either mature or overripe [55,56]. Dried fruits and nuts have been seen to occasionally contain this particular species, and there is a lack of empirical evidence supporting its ability to sustain or grow a noteworthy population while in storage [56]. Last but not least, *Amyelois transitella* is a polyphagous species that consumes grapes, citrus, almonds, pistachios and many other relative commodities. *A. transitella* inhabits a variety of climates in North, Central, and South America [57]. Most infestations from larvae take place in storage with harvested products from the field, but the species cannot reproduce under storage conditions. Although intercepted in Italy and in Austria, this species is not believed to be present within the European Union (EU) [58]. Nevertheless, due to phytosanitary restrictions, and yield and quality losses, *A. transitella* satisfies the EFSA-amendable criteria for its classification as a future quarantine pest in the EU [58,59].

Dried fruits exhibit a heightened susceptibility to infestations by mites and numerous investigations have been conducted on mite contamination in dried fruits and related commodities [25,60,61]. These studies have been carried out by analyzing samples collected from several geographical regions across the globe. For instance, a notable recorded contamination level reaching 15,000 mites per kilogram of dried apricots was reported in Turkey [62]. Tao et al. [63] collected a total of 12 mite species, belonging to 6 families and 10 genera from the 49 samples of dried fruit stores and warehouses in China. In that study, the authors found that the dominant mite species were the dried fruit mite, *Carpoglyphus lactis* (L.) (Acari: Astigmata), the cheese mite, *Tyrophagus putrescentiae* (Schrnak) (Sarcoptiformes: Acaridae), the flour mite, *Acarus siro* L. (Astigmatina: Acaridae), and *Caloglyphus berlesei* (Michael) (Sarcoptiformes: Acaridae) [63]. Another research reported that nearly 77% of the collected samples of dried apricots, figs, and chestnut fruits in Egypt were infested with six species of mites, namely *C. lactis*, *T. putrescentiae*, *Blattisocius keegani* (Fox) and *B. dentriticus* (Berlese) (Mesostigmata: Blattisociidae), *Cheyletus malaccensis* (Oudemans) (Prostigmata: Cheyletidae) and *Tarsonemus sp*. (Heterostigmatina: Tarsonemidae) [64]. Compared to other stored-product pests, mites have a greater dependence on elevated humidity levels in order to facilitate their rapid development and population growth, while they are very susceptible to dry conditions due to dehydration. Nevertheless, they exhibit economic significance due to their ability to produce allergens and act as vectors for several kinds of fungi and bacteria [31,65,66]. Yet, information related to mite infestation within the food production and marketing chains is commonly considered as market-sensitive, given that the EU and the United States (US) authorities have established strict regulations regarding mite infestation in stored products, with a zero- or near-zero-tolerance policy [67]. As a result, manufacturers implement strict measures to ensure the confidentiality of such information and data related to direct mite contamination in food products within industrial and retail settings [25].

**Table 1:** Insect and mite species that can infest stored dried fruits [28,63,64,68].

**Coleoptera**	
Dried fruit beetle	*Carpophilus hemipterus* L.
Confused sap beetle	*Carpophilus mutiatus* Erichson
Corn sap beetle	*Carpophilus dimidiatus* (F.)
Australian sap beetle	*Carpophilus davidsoni* Dobson
Pineapple beetle	*Carpophilus humeralis* (F.)
Rust red flour beetle	*Tribolium castaneum* Herbst
Drugstore beetle	*Stegobium panaceum* L.
Cigarette beetle	*Lasioderma serricorne* F.
Sawtoothed grain beetle	*Oryzaephilus surinamensis* L.
Merchant grain beetle	*Oryzaephilus mercator* Fauve
Small darkling beetles	*Blapstinus* spp.
Hairy fungus beetle	*Typhaea stercorea* (L.)
Yellow nitidulid	*Haptoncus luteolus* (Erichson)
Leadcable borer	*Scobicia declivis* (LeConte)
Date-stone beetle	*Coccotrypes dactyliperda* (F.)
-	*Gonocephalum pusillum* F.
**Lepidoptera**	
Raisin moth	*Ephestia figuiela* Gregson
Codling moth	*Cydia pomonela* L.
Almond moth	*Ephestia cautela* Walker
Indian meal moth	*Plodia interpunctela* Hubner
Tobacco moth	*Ephestia elutella* Hubner
Rice moth	*Corcyra cephalonica* Stainton
Navel orangeworm	*Amyelois transitelfa* Walker
Raisin moth	*Ephestia figuiela* Gregson
Codling moth	*Cydia pomonela* L.
Almond moth	*Ephestia cautela* Walker
**Mites**	
Dried food mite	*Carpoglyphus lactis* (L.)
Cheese mite	*Tyrophagus putrescentiae* (Schrnak)
Flour mite	*Acarus siro* L.
-	*Cheyletus malaccensis* (Oudemans)
-	*Blattisocius keegani* (Fox)
-	*Blattisocius dentriticus* (Berlese)
-	*Caloglyphus berlesei* (Michael)
-	*Tarsonemus* sp.

## 3. Fungi and Mycotoxins Commonly Found in Dried Fruits

Mycotoxins, a noteworthy cluster of low-molecular-weight compounds (below 1000 Da), may be deemed the secondary metabolites produced by strains of filamentous moulds, usually belonging to the genera of *Aspergillus*, *Fusarium*, *Penicillium*, and *Alternaria* [69]. The types of mycotoxigenic fungi can be divided into three wide groups, according to Miller [70]:

(i) Hydrophilic plant pathogenic fungi such as *Fusarium graminearum* Schwabe, named “field fungi”;
(ii) Saprophytic and thermophilic fungi such as *Fusarium proliferatum* (Matsushima) Nirenberg or *Aspergillus flavus* Link, considered as “intermediate microflora”;
(iii) Xerophilic moulds that colonize products after the harvest at favourable product water content, such as *Penicillium verrucosum* Dierckx or *Aspergillus ochraceus* Wilhelm, so-called “storage microflora” [71,72].

The production of dried fruits for human consumption is significant, with raisins, sultanas, figs, apricots, and dates being the most important [8]. The contamination of these fruits with mycotoxins can start from the tree, further escalating during harvesting and sun drying, and persisting during storage. The warm climate in which these fruits are cultivated makes them susceptible to mycotoxins, with the most common in dried fruits being aflatoxins, OTA, and *Alternaria* toxins. *Aspergillus flavus* and/or *A. parasiticus* are the primary producers of aflatoxins in figs. Moreover, these mycotoxins were detected in various dried fruits such as raisins, figs, apricots, and dates [73]. Abdel-Sater and Saber [74] discovered that *Aspergillus* was the foremost isolated genus in date samples, exhibiting contamination levels of up to 100%. Conversely, *Penicillium* was less commonly isolated, with only 30% of the data samples exhibiting contamination. The most abundant species that was found in contaminated dates was *Aspergillus niger* van Tieghem [75]. Furthermore, according to Toma and Rajab [76], the development of certain species of *Aspergillus* (*A. niger*, *A. carbonarius* and *A. ochraceus*) is facilitated by the concentration of high sugar and low water activity in dried fruits, due to their xerophilic nature. *Aspergillus niger* was observed to be the most prevalent fungus in the majority of dried fruit samples [76]. In addition to that, dried raisins are prone to the growth of fungi and the consequent production of mycotoxins due to their elevated levels of moisture and sugar content, as reported by Alghalibi and Shater [77]. In the research conducted in 2006 by El Khoury et al. [78] in Lebanon, the fungal species responsible for aflatoxin production in grapes was investigated, with their results demonstrating that *A. flavus* is the primary source of aflatoxin. According to their analytical results, Juan et al. [79] revealed that samples of dried raisins had a 20% frequency of AFB1 contamination. The values of AFB1 varied between 3.2 and 13.9 µg/kg. The EU standards for AFB1 established a maximum limit of 2 µg kg^−1^, which was surpassed by all positive samples. The current survey showed the presence of AFs in dried raisins, despite numerous studies suggesting that they do not appear to be a suitable substrate for A. flavus growth and AFs synthesis.

Dried fruits are prone to the emergence of mould and mycotoxin contamination, subject to the procedures and conditions of preservation. The consumption of dried fruits contaminated with mycotoxins could result in unfavourable health consequences [16,80]. Based on the annual reports of RASSF from 2018 to 2020, it has been observed that dried fruits imported by EU countries have received more than 50 notifications annually due to the detection of high levels of aflatoxins and/or OTA [81,82,83]. In general, mycotoxins were the third most notified hazard category (485 notifications with a 10.5% increase compared to 2021).

During the period between 2017 and 2021, a comprehensive sum of 325 notifications of the Rapid Alert System for Food and Feed (RASFF) pertaining to dried figs were officially released. Among these notifications, it was observed that AFs accounted for a substantial 82.8% (269 notifications), thus establishing it as the primary source of mycotoxin contamination. Following closely behind, OTA emerged as the second most prevalent source with 34 notifications, representing 10.5% of the total. The remaining 22 notifications, constituting 6.7% of the total, were associated with various other hazards, including insects, sulphites, and pesticides [84,85].

Combinations of mycotoxins, including OTA and AFB1, were found in dried figs from Turkey, with levels as high as 63 ng g^−1^. These findings highlight the potential health risks associated with the consumption of contaminated dried fruit products [85]. The examination of the mycological composition of date fruits sold in local markets in Saudi Arabia revealed the existence of AFs and OTA, which can be attributed to the production of *A. flavus*, *A. niger*, and *A. terreus*, as reported by Gherbawy et al. [86]. Dried fruits are highly susceptible to contamination by mycotoxins such as AFB1, AFB2, AFG1, and AFG2 throughout the entire process, from harvesting to marketing [87]. Nonetheless, a recent study conducted in South Punjab, Pakistan, by Naeem et al. [88] reported that the growth of the mycotoxigenic mould in dry fruits stored at 4 °C and closed glass containers was inhibited, as determined by margin of exposure assessments. This study highlights the potential impact of proper storage techniques on reducing the risk of mycotoxin contamination in dried fruits. Dried plums (*n* = 27) and other dried fruits (dried vine fruits, dried figs, dates, and dried apricots) were collected in 2012–2013 from different markets in Tunisia and Spain and tested for 16 mycotoxins. Dried plums had the lowest contamination rate (25.9%) for the targeted mycotoxins among all the dried fruit samples tested, while prune samples, had the highest levels of AFG2 (15.6 µg kg^−1^), HT-2 toxin (10 µg kg^−1^), and diacetoxyscirpenol (DAS) (135 µg kg^−1^). This study also indicated that AFG2, OTA, and HT-2 toxin co-occurred in seven dried plum samples, with four of them being purchased from Tunisia and three from Spain [89].

Ochratoxin A is produced by various *Aspergillus* species, primarily *A. ochraceus*, *A. niger*, and *A. carbonarius*, along with certain *Penicillium* species [90,91] (Table 2) and is considered as a significant nephrotoxin with teratogenic, hepatotoxic, and immunosuppressive properties, and is designated as a Group 2B carcinogen by the IARC [92]. Aflatoxins are well-documented and among them, AFB1 is widely recognized for its toxicity. This particular toxin is known to be teratogenic, carcinogenic, mutagenic, and immunosuppressive, and IARC has classified it as a Group 1 carcinogen [92]. Janati et al. [93] conducted research for the determination of OTA and AF levels in dried prunes, wherein they demonstrated that approximately 13.33% of the prune samples were found to be positive for aflatoxin presence. Numerous reports have indicated that dried fig fruits are considered a high-risk commodity with respect to toxigenic fungi and their mycotoxins. The most frequently reported toxigenic fungi include *Aspergillus* section *Nigri*, *Fusarium* spp., *Aspergillus* section *Flavi*, and *Penicillium* species [94]. In Turkish dried figs, several other genera of moulds were identified, including species of *Acremonium*, *Byssochlamys*, *Cladosporium*, *Trichoderma*, *Mucor*, and *Scopulariopsis* [95]. Regarding products coming from grapes, the proliferation of *A. carbonarius* and *A. niger* is a phenomenon observed in post-harvested grapes, which ultimately leads to the formation of OTA during the process of sun drying. It should be noted that black Aspergilli are capable of sustained proliferation and production of OTA until grapes have achieved a water activity level of less than 0.80 [96,97]. Tjamos et al. [98] documented that, despite the prevailing *Aspergillus* spp. found in Corinth raisins and grape cultivars used for wine production in Greece being primarily from the *A. niger* aggregate, the isolates of *A. carbonarius* demonstrated the highest level of efficiency in producing OTA. The natural occurrence of OTA has been reported in dried apricots and dried plums (prunes) [99,100]. Furthermore, the co-occurrence of OTA and citrinin in coconut products was reported from India [101]. In addition, high rates for OTA have been reported by other research groups: 80–84% at 2, 5 and 10 ng g^−1^ [102], 82.4% at 10 ng g^−1^ [103] and from 80% to 85% at 1, 7.6 and 29.6 ng g^−1^ [98] in dried fruits. Furthermore, MacDonald et al. [104] reported low levels of OTA in currants, sultanas, and raisins, ranging from 69% to 74%.

Reports from the Rapid Alert System for Food and Feed (RASFF) illustrate that the presence of OTA in dried grapes poses a notable risk, particularly in regions such as Turkey, Iran, Uzbekistan, Pakistan, Greece, and South Africa. An investigation was conducted by Fanelli et al. [105] to quantify the extent of OTA contamination in dried grapes sourced from diverse geographical areas, including Chile, China, Iran, Turkey, South Africa, and the United States. The findings revealed that the highest average contamination levels were observed in samples obtained from Turkey, measuring at 3.1 µg kg^−1^ [105]. Data regarding the presence of OTA contamination in dried grapes consumed within the EU is also accessible. A total of 562 findings pertaining to the incidence of OTA in dried grapes were reported by four countries (France, Germany, Greece, and the United Kingdom). In France, the frequency of OTA contamination in dried grapes was measured at 46.2%, while in Germany, it was 94.3%, in Greece, it was 54.9%, and in the United Kingdom, it was 91.1%. The maximum recorded levels of OTA contamination in dried grapes were 4.3 µg kg^−1^ in France, 21.4 µg kg^−1^ in Germany, 16.5 µg kg^−1^ in Greece, and 53.6 µg kg^−1^ in the United Kingdom [106]. In a recent review, González-Curbelo and Kabak [88] reported that in surveys conducted from 1998 to the present, in a total of 17 countries, the presence of OTA was detected in 2450 out of 3117 samples of dried grapes, accounting for a prevalence rate of 78.6%. It is noteworthy to mention that Greece, among the 17 countries under investigation, exhibited both the highest incidence rate (up to 100%) and the most significant level of contamination in dried grapes, reaching 138.3 µg kg^−1^.

*Alternaria* spp. (Table 2) have the capability to produce mycotoxins that have the potential to contaminate food and feed products [107]. Alternariol Monomethyl Ether (AME), one of the most frequently produced mycotoxins by *Alternaria*, has been identified a variety of fruits and their by-products, including apple juice, grape juice, tomato sauce, and dried fruits [108]. In a survey conducted in the Netherlands by Lopez et al. [109], AME was detected in selected food categories such as dried figs, sunflower products, and tomato products.

Patulin, a mycotoxin produced by various *Penicillium* species including *P. expansum*, *P. chrysogenum*, and *P. nordicum*, remains an important contaminant due to its potential risks to human health (Table 2). In this regard, it is important to acknowledge the fact that patulin has the potential to undergo degradation during the course of food processing or storage, thereby leading to the creation of alternative toxic substances [110]. Moreover, patulin has the capacity to interact with other compounds within food, thereby potentially influencing its bioavailability and toxicity. Hence, although patulin may not be classified as a masked mycotoxin, it remains crucial to exercise vigilance and exert control over its presence in food products [111]. *Penicillium expansum* is the source of blue mould in apples and apple-related products and is the most prevalent and significant patulin producer [112]. Patulin was primarily detected in apples, specifically in decayed portions of the fruit. Numerous surveys have been conducted worldwide so far to examine the extent of patulin contamination in apples and apple-based concentrates [113,114], with limited research conducted on dried fruits such as dried figs [115]. For instance, Karaca et al. [116] identified the presence of patulin in dried figs, exhibiting patulin quantities ranging from 40 to 150 ng g^−1^. These levels fall within the milligram per kilogram range, suggesting a significant prevalence of fungal contamination.

Fumonisins pertain to mycotoxins that are primarily synthesized by species originating from the genus *Fusarium*, with a particular emphasis on *F. verticillioides* and *F. proliferatum* [117] (Table 2). Limits for total fumonisins B1 (FB1) and B2 (FB2) have been set for cereals and cereal-based products [19]. Among the fumonisin derivatives, FB1 is the most common one and constitutes about 70–80% of the total fumonisin content of *F. verticilloides* cultures and naturally contaminated foods. Additionally, their synthesis has been observed in selected members of the *Aspergillus* species, namely *A. niger*, and on rare occasions, *A. welwitschiae*, up until the present time. Szecsi et al. [118] reported that Aspergilli produce much smaller quantities of fumonisins than Fusaria. A research study demonstrated that *Aspergillus* section *Nigri* isolates obtained from dried fig samples were capable of producing FB2 in the culture medium and in figs [119]. In Greece, the presence of FB2 was detected in 12 out of 42 raisin samples (29%), with concentrations ranging from 7.1 to 25.5 µg kg^−1^, and out of these, 6 samples exhibited the simultaneous occurrence of OTA [120]. Another research study reported the occurrence of FB2 in Turkish dried grapes, in a limited number of samples, specifically 1 out of 60 (1.7%) at a level of 8.8 µg kg^−1^ [121]. Moreover, a study examined the mycobiota and fumonisin contamination of dried vine fruit samples, and found that black Aspergilli, specifically *Aspergillus niger* and *Aspergillus awamori*, were responsible for fumonisin contamination in these products [122]. It has also been demonstrated that the fumonisin content in the samples contaminated by potential fumonisin-producing black *Aspergilli* ranged from 4.55 to 35.49 mg kg^−1^ [123]. These findings suggest that dried figs can be affected by fumonisin mycotoxins, which are produced by certain fungal species, particularly *Aspergillus* section *Nigri*, and pose a potential threat to food safety [122]. In a study conducted by Susca et al. [124], 48 strains belonging to *Aspergillus* section *Nigri*, isolated from grapes and raisins, were thoroughly investigated to assess the presence of the *fum8* gene—a key gene in the fumonisin biosynthetic pathway—and its correlation with FB2 production. Of the 48 strains examined, fum8 was detected in 11 strains of *A. niger*. Notably, nine of these fum8-positive strains demonstrated the ability to produce FB2, suggesting a potential association between the presence of this gene and mycotoxin biosynthesis [124]. Additionally, in another study, it was observed that among a larger pool of 66 *A. niger* strains, 4 of them were identified as producers of both FB2 and FB4 when found in raisins [125].

The existing research has also indicated that mycotoxin contamination in dried fruits can extend beyond the types that have been previously identified. For example, other mycotoxins, such as FB2 [121], HT-2 [84], T-2 [126], enniatins [126] and diacetoxyscirpenol (DAS) [89,127], have been identified. For instance, prune samples, along with various other dried fruits, exhibited the most elevated concentrations of AFG2 (15.6 µg kg^−1^), HT-2 toxin (10 µg kg^−1^), and DAS (135 µg kg^−1^). The investigation further demonstrated that AFG2, OTA, and HT-2 toxin were found concurrently in seven samples of dried plums, four of which originated from Tunisia and three from Spain [128].

Mycotoxins can be produced under various conditions, depending on temperature, water activity, and pH, when certain fungi are present (Table 2). The occurrence of mycotoxins is more frequent in areas with a hot and humid climate, which is favourable for the growth of moulds [129]. Earlier surveys have been conducted in numerous countries regarding wine and grape juice derived from tainted grapes for the purpose of assessing the presence of OTA [130].

**Table 2:** Mycotoxins and producing species in dried fruits, their optimal temperature growth conditions, and health implications.

Mycotoxin	Fungi	Range of Temperature (°C)	Reference	Symptoms/Toxicology
Aflatoxins	*Aspergillus flavus* *Aspergillus parasiticus*	10–48 12–42	[131]	Binds to guanine (DNA adduct)/carcinogenic, mutagenic, teratogenic, hepatotoxic [132]
Ochratoxin A (OTA)	*Aspergillus ochraceus* *Aspergillus niger* *A. carbonarius* *Penicillium verrucosum*	10–40 6–47 10–40 0–31	[133]	Blocks protein synthesis/mutagenic, teratogenic, neurotoxic, hepatotoxic, nephrotoxic, immunotoxic [134]
Fumonisins	*Fusarium verticilloides* *Fusarium proliferatum*	15–35	[135]	Carcinogens, neurotoxic, neural tube defects, genotoxic [136]
Alternariol Monomethyl Ether Alternariol	*Altenaria* spp.	5–30	[108]	Genotoxic and carcinogenic in animal studies, internal hemorrhage [137]
Patulin	*Penicillium expansum*	25	[112]	Nausea, vomiting, and diarrhea, immunotoxicity and genotoxicity [137]

## 4. The Storage Ecosystem: Dynamics of Infestation and Ecological Interactions in Stored Products

Τhe storage environment, characterized by its abundant food sources and stable environmental conditions, presents an immensely attractive habitat for a diverse range of organisms to grow and infest the product while stored [30,34]. This storage ecosystem typically consists of three predominant biotic communities: the insect community, encompassing species responsible for food loss; the fungal community, comprising species capable of thriving under certain environments; and the mammal communities, in which rodents and mice are mostly associated [138,139]. Environmental factors including storage structures (silos, bins, bags, etc.), temperature, humidity, and gases have a significant influence on niche spaces and successional events of these biotic communities [140]. Sinha et al. [141] conducted an eight-year study testing the interaction of 32 variables, involving both biotic and abiotic factors, in two 13.6-ton stored wheat bulks. Based on their findings, the key variables of importance in this longitudinal investigation were grain evaluations, particularly with regard to the duration of storage, as impacted by fungal and insect infestations, while temperature and moisture also exerted a considerable influence on the quality and viability of stored grains [141]. Although this study focused on wheat, the ecological principles and environmental interactions it describes are likely broadly applicable to dried fruit storage, where comparable biotic and abiotic factors influence product quality and safety.

Stored-product insects have the ability to assume many functions within these ecological structures [142]. The infestation often begins with the emergence of primary feeders such as the rice weevil, *Sitophilus oryzae* (L.), the granary weevil, *Sitophilus granarius* (L.), the maize weevil, *Sitophilus zeamais* Motschulsky (Coleoptera: Curculionidae), and the lesser grain borer, *Rhyzopertha dominica* (F.) (Coleoptera: Bostrychidae). These species rank among the most destructive stored-product insects due to their highly specialized mouthparts, which enable them to penetrate and infest intact products, including those that are securely packaged [143]. For instance, weevils possess elongated snouts, or rostrums, which are used to bore into intact fruits or grains [144]. Females then deposit eggs into these cavities, with each female capable of laying 200–400 eggs, each housed individually [144]. Inside this protective environment, the larvae develop by consuming the internal contents of the fruit or grain, effectively hollowing it out while remaining concealed. In a similar manner, *R. dominica* uses its strong mandibles to chew through the surface of the stored product [145]. Unlike weevils, it lays eggs on the surface of the fruit, and the larvae burrow inside immediately after hatching. Both types of insects complete their entire life cycle—from eggs to larvae, pupae, and adults—within the infested product. This exclusive internal development makes early detection extremely challenging, as the damage remains hidden until the infestation has progressed significantly [143]. By the time signs of infestation, such as brittle shells or powdery residues, become visible, much of the product’s internal content has been consumed. The result is a hollowed, degraded product that is unsuitable for consumption or sale, leading to significant economic losses [146]. A successional progression of the infestation occurs by the subsequent colonization by secondary pests, such as the rusty grain beetle, *Cryptolestes ferrugineus* (Stephens) (Coleoptera: Cucujiidae) and *O. surinamensis*, species that cannot penetrate intact fruits but thrive on the damaged products left by primary feeders [145]. While the quality of grain progressively declines due to these secondary pests, scavengers such as species of the Dermestidae and Mycetophagidae families often appear later in the infestation cycle, feeding on residues, debris, or mould-covered materials [28,34,146].

The spoilage of stored products is markedly accelerated by the formation of damp heating zones, commonly referred to as “hot spots”. These are created by the metabolic respiration and active growth of insects, which generate localized increases in temperature and moisture, ideal conditions for the proliferation of fungal communities [147,148]. For this reason, fungi associated with storage typically emerge concomitantly with or after insect infestations, as insects not only create entry points for fungal spores but also modify the microenvironment to favour fungal growth [149]. The growth of fungi, in turn, generates additional heat and moisture, creating a feedback loop that rapidly escalates environmental changes within the affected area. Hot spots are also associated with the accumulation of gases (e.g., CO_2_ from respiration) and volatile organic compounds (VOCs), which further attract certain pests and microbes [150]. In conclusion, the interplay between insects and pathogens in storage hot spots, driven by their distinct environmental preferences, fosters complex ecological dynamics that significantly influence infestation severity and contribute to the multifaceted nature of storage-related spoilage.

## 5. Synergistic Effects Between Stored-Product Insects and Post-Harvest Pathogen

Despite the intricate nature of the association between insects and pathogens, various outcomes can be observed in scenarios that involve the interplay between these two groups [151]. Indicatively, certain insect species serve as vectors of storage fungi and bacteria [152], whereas others function as exterminators [153,154]. Conversely, certain fungi promote the population growth of insects by feeding and/or attracting them [155,156,157], while others repel them by secreting substances (including mycotoxins) that are toxic to insects [23]. The association and synergistic interactions between insects and fungi have been thoroughly documented in relation to stored products [22,158,159,160]. It is noteworthy that the research is specifically focused on insect species that possess the ability to carry and spread spores of the three major genera of fungi that produce mycotoxins, i.e., Aspergillus, Fusarium, and Penicillium [21], via their exoskeletons or intestines [22,23].

Multiple studies have demonstrated that the presence of the major insect pests of dried fruits can contribute to the significant dispersal and growth of fungal infestations and subsequent contamination of stored products [148]. For instance, *Carpophilus* spp. demonstrates notable adaptations facilitating the transmission of mycotoxigenic fungus, by vectoring the fungal spores within their bodies or through their gastrointestinal tracts. While these toxins can be deadly to insects, *Carpophilus* spp. have a far higher tolerance to aflatoxicosis compared to other insect pests, ranging from 10- to 100-fold [161,162,163,164,165]. Likewise, a plethora of studies have illustrated the substantial role of *T. castaneum* in the spread of fungal contaminants, increasing the presence of mycotoxins in several stored products [166,167]. Thus, since the mycotoxin contamination in dried fruit is a widely observed phenomenon [168,169], it is reasonable to hypothesize that there are synergistic interactions between mycotoxigenic fungi and insect pests of dried fruits. Nevertheless, there is a scarcity of published data regarding the synergistic association between insects and fungi when it comes to infestation on dried fruits, in contrast to the literature on other stored commodities like wheat and maize [22,170,171].

Similar to insects, studies have documented that mites obtain nutritional value through their symbiotic association with mycotoxin-producing fungi, specifically *Aspergillus* spp. and *Penicillium* spp., as reported by Aucamp [172], Franzolin et al. [173], and Hubert et al. [174]. For instance, choice tests conducted by Thomas and Dicke [175] indicated that *A. siro* often exhibited a great attraction and feeding response for *Penicillium camemberti* Thorn, *A. flavus*, and *A. repens* (Corda) Debary. This phenomenon has been corroborated by other studies, which observed a decrease in the number of colonies of some species of *Aspergillus* and *Penicillium* in the presence of *A. siro* [176]. The population reduction may be attributed to the secretion of fungitoxic chemicals by the mites or alternatively, to the mites feeding on the fungi [177,178,179]. Moreover, stored-product mites, which frequently consume fungus found in stored grain such as *Alternaria*, *Aspergillus*, *Cladosporium*, and *Penicillium*, have been identified as carriers of these fungi [180]. For instance, the cheese mite, *T. putrescentiae*, has been characterized as a vector for eight fungal species, including *Aspergillus clavatus* Desm. and *Penicillium citrinum* Thom, among others [159]. Although instances of mycotoxicosis have been noted for *T. putrescentiae* [181], the mite has the ability to develop and reproduce by consuming a diet composed of mycotoxin-producing strains of *Fusarium* spp. [182,183,184]. It is important to acknowledge that most of the literature in this field is relatively outdated, and additional investigation is necessary to modernize the data.

Based on the above, a direct correlation between the presence of stored-product mites and the risks associated with fungal infection in dried fruit can be considered as self-evident and is particularly important in the case of mycotoxigenic strains. Thus, the identification of the species within general successional patterns can potentially serve as an indicator of the grain quality [184,185]. Considering the emphasis placed on increasing awareness within the industry and the general public regarding the potential hazards of arthropod-borne food contamination [31], the detection of insects and mites in dried fruits is commonly perceived as a potential indicator of health-related issues pertaining to food quality and safety.

## 6. Climate Change and Mycotoxins

Climate change (CC) has had a significant impact on the whole agricultural supply chain in recent years. To this end, CC similarly impacts the spread of stored-product pest insects, in a manner analogous to that of field crops and orchard pests [186]. A crucial aspect of CC is the increase in global temperature, which can lead to a rise in atmospheric humidity. The transition towards higher temperatures and increased humidity conditions facilitates the growth of insect pests and moulds [142]. Furthermore, warmer conditions may have a direct impact on the occurrence and spread of storage pests in cooler areas, such as northern Europe [187]. Consequently, a shift in the population dynamics and distribution of pests that infest stored products may increase the potential risk of mycotoxin contamination.

Currently, there is a need for more detailed information on the mycotoxin presence in dried fruits as far as this is linked to factors related to CC. Germination, growth, and mycotoxin production by the responsible genera (*Aspergillus*, *Fusarium*, *Penicillium*, and *Alternaria*) are subject to modifications in the levels of water, temperature, and CO_2_, which potentially exert modulations influencing the mycotoxin contamination of dried fruit products [188]. Additionally, alterations in the prevalence of mycotoxins among these species may also be manifested [189,190]. However, there has been a notable lack of research aimed at investigating the impact of various environmental conditions, particularly those related to CC, on the production and detection of mycotoxins. Over the past thirty years, an amount of research has been dedicated to analyzing the effects that temperature-related variables exert on the proliferation and synthesis of mycotoxins by a multiple array of mycotoxigenic fungi [191,192]. Nevertheless, it should be noted that certain phenomena, such as extreme drought, desertification, and oscillations in moisture levels, are expected to elicit consequences on the life cycles of mycotoxigenic fungi [14]. As noted, significant alterations in plant physiology may alter the pathogen–host dynamic in the context of climate change [14,15]. As such, it is imperative to conduct research on whether these changes will result in modifications to plant protection mechanisms, and whether this will lead to induced or reduced contamination. Under CC scenarios, there may be changes in the proportion of free and masked mycotoxins, thereby necessitating more comprehensive analyses in the immediate future.

## 7. Legislation

Mycotoxin legislation in the European Union (EU) has undergone significant development in the last twenty years. The EU has established maximum levels of mycotoxins allowed in human food through Legislation EC No 1881/2006 and No 1126/2007 [193]. These regulations cover mycotoxins such as aflatoxins, fumonisin, OTA, patulin, trichothecenes, and zearalenone [193]. The EU mycotoxin regulatory developments have been influenced by various European organizations and programmes, including the European Food Safety Authority, the Scientific Cooperation on Questions relating to Food, and the Rapid Alert System for Food and Feed. The EU has also established a Community Reference Laboratory for Mycotoxins and mandated the European Standardization Committee to develop methods for mycotoxin analysis in food [194]. These efforts have contributed to the harmonization of mycotoxin regulations within the EU. The European Union has established official regulations and guidelines for maximum levels of OTA and aflatoxins in foodstuffs such as cereals, coffee, dried fruits, wine, and grape juices [187]. However, in numerous developing countries, particularly in Africa, where traditional agricultural practices play a significant role in food production and consumption, the implementation of such measures would pose significant challenges [195]. There is increasing interest among researchers in determining the contamination levels of mycotoxins and more specifically of OTA and aflatoxins, and among regulatory agencies to establish new or replace old mycotoxin limits for food commodities due to its widespread occurrence, increasing health risk concerns, and the development of new sensitive analytical methods.

The Commission of the European Communities has released European Legislation pertaining to certain mycotoxins found in dried fruit. According to these regulations, the maximum level (ML) of OTA in dried vine fruit (such as currants, raisins, and sultanas) is 8 ng g^−1^. The MLs of 2 and 4 µg kg^−1^ for AFB1 and the sum of AFs (AFT, i.e., AFB1 + AFB2 + AFG1 + AFG2), respectively, in dried fruits designated for human consumption have been established by the European Commission. However, if the dried fruit is to undergo sorting or other physical treatment, the maximum allowable levels are 5 ng g^−1^ for AFB1 and 10 ng g^−1^ for total aflatoxins [17,18].

## 8. Conclusions and Future Perspectives

This review serves to bring attention to the noteworthy danger presented by stored-product insects, fungi, and mycotoxins in dried fruits. The infestation of pests and the concentration of mycotoxins in dried fruits are subject to change from year to year, closely linked to the fluctuations in climate conditions. In order to minimize the adverse effects of climate change on the synthesis of mycotoxins in dried fruits, it is imperative to adopt a comprehensive approach that incorporates both mitigation and adaptation strategies. This necessitates the implementation of meticulous storage conditions, which encompasses the precise regulation of temperature, humidity, moisture levels, packaging, and pest control measures, all while adhering to the principles of good manufacturing practices. In this context, the presence of certain stored-product insects can serve as a reliable indicator of the potential development of fungi during storage, processing, and transportation, and the concomitant mycotoxin contamination.

The occurrence of outbreaks and recalls resulting from contamination by foodborne pathogens, in addition to concerns related to mycotoxins, emphasize the necessity for further research in the management of these identified pests, moulds, and mycotoxins. Over the last decade, there has been notable progress made in the design and effectiveness of contemporary drying techniques, such as vacuum drying, freeze drying, microwave drying, infrared drying, dielectric drying, supercritical carbon dioxide drying, and low-pressure superheated steam drying. Additionally, proper storage and packaging techniques can help control pest infestation in dried fruits. Sealing dried fruits airtightly with a deoxidation material packed together, in a packaging material with specific moisture permeability, can help control insect pests without compromising the quality of the dried fruits. Furthermore, controlling the quality of dried fruits on the market is crucial for consumer safety, including monitoring antioxidant activity, contamination with toxic metals, and the presence of mycotoxins.

## Data Availability

No new data were created or analyzed in this study.
